# Cell Density- and Quorum Sensing-Dependent Expression of Type VI Secretion System 2 in *Vibrio parahaemolyticus*

**DOI:** 10.1371/journal.pone.0073363

**Published:** 2013-08-15

**Authors:** Li Wang, Dongsheng Zhou, Panyong Mao, Yiquan Zhang, Jun Hou, Yan Hu, Jin Li, Shaojie Hou, Ruifu Yang, Runhua Wang, Jingfu Qiu

**Affiliations:** 1 School of Public Health, Chongqing Medical University, Chongqing, China; 2 State Key Laboratory of Pathogen and Biosecurity, Beijing Institute of Microbiology and Epidemiology, Beijing, China; 3 Beijing 302 Hospital, Beijing, China

## Abstract

**Background:**

*Vibrio parahaemolyticus* AphA and OpaR are the two master quorum sensing (QS) regulators that are abundantly expressed at low cell density (LCD) and high cell density (HCD), respectively, with a feature of reciprocally gradient production of them with transition between LCD and HCD. The type VI secretion system 2 (T6SS2) gene cluster can be assigned into three putative operons, namely VPA1027-1024, VPA1043-1028, and VPA1044-1046. T6SS2 contributes to adhesion of *V. parahaemolyticus* to host cells.

**Methodology/Principal Findings:**

OpaR box-like sequences were found within the upstream promoter regions of all the above three operons, while none of AphA box-like elements could be identified for them. The subsequent primer extension, LacZ fusion, electrophoretic mobility shift, and DNase I footprinting assays disclosed that OpaR bound to the promoter regions of these three operons to stimulate their transcription, while AphA negatively regulated their transcription most likely through acting on OpaR. This regulation led to a gradient increase of T6SS2 transcription with transition from LCD to HCD.

**Conclusions/Significance:**

*V. parahaemolyticus* OpaR and AphA positively and negatively regulate T6SS2 expression, respectively, leading to a gradient elevation of T6SS2 expression with transition from LCD to HCD. T6SS2 genes are thus assigned as the QS regulon members in *V. parahaemolyticus*.

## Introduction

**Figure 1:**
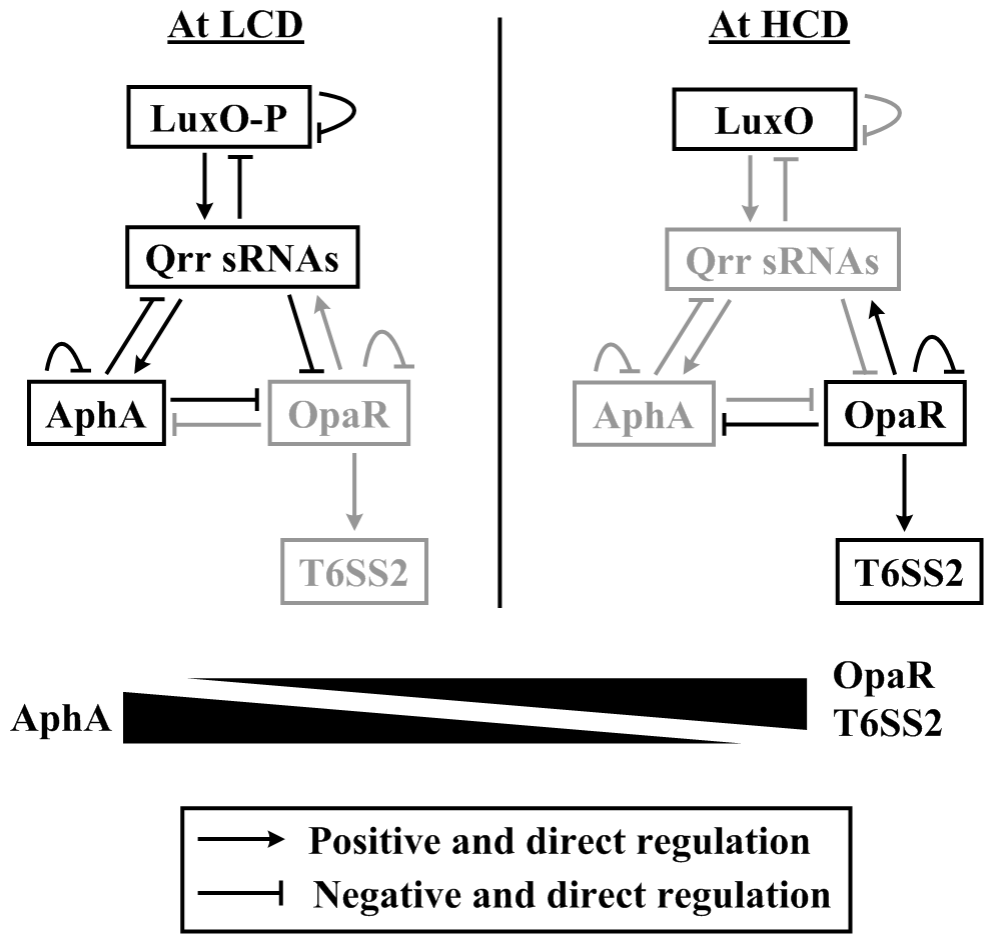
Action of *V. parahaemolyticus* QS systems. The regulatory associations between LuxO, Qrr sRNAs, AphA, and OpaR were summarized with the integration of relevant observations in *V. parahaemolyticus* [4,5] and closely related *V. harveyi* [3,32–40]. The grey fonts denoted the inhibited production of relevant proteins or the cease of relevant regulatory cascades. At LCD, low concentrations of autoinducers lead to phosphorylation of LuxO (LuxO-P), and LuxO-P activates expression of Qrr sRNA genes [32,33]. Redundant Qrr sRNAs promote AphA translation and, meanwhile, inhibit OpaR translation [34–36]. AphA further represses *opaR* transcription [3,5]. Overproduced AphA feeds back to inhibit transcription of *qrr2-3* and its own gene [3,5]. In addition, over-production of Qrr sRNAs and LuxO-P triggers three additional feedback regulatory loops: i) LuxO-P represses transcription of its own gene, ii) Qrr sRNAs inhibits *luxO* translation, and iii) Qrr sRNAs repress translation of *luxMN* encoding the membrane-anchoring autoinducer-binding receptor protein LuxM and its cognate receptor LuxN [37–39]. The above feedbacks will contribute to control the LuxO-P, Qrr and AphA levels within the physiological states. At HCD, high concentrations of autoinducers reverse the phosphate flow in the circuit, leading to dephosphorylation of LuxO. Dephosphorylated LuxO is inactive as a regulator, leading to cessation of Qrr sRNA production; thus, there is no production of AphA but OpaR translation occurs [34,35]. OpaR in turns represses *aphA* transcription [4,40] but stimulates T6SS2 transcription (this study), and it also feeds back to inhibit its own expression [4,40]. OpaR is also able to activate the *qrr2-4* transcription, leading to rapid down-regulation of *opaR* [4,40]; this OpaR-*qrr* feedback dramatically accelerates transition from HCD to LCD, but it has no effect on QS behaviors at steady-state LCD or HCD [41]. Taken together, there is the reciprocal gradients of cellular AphA and OpaR levels during transition between LCD and HCD, and AphA and OpaR act as the master QS regulators at LCD and HCD, respectively. In addition, T6SS2 transcription enhances in a gradient manner with transition from LCD to HCD, which is coordinately controlled by AphA and OpaR.

Quorum sensing (QS) systems are widely distributed in *Vibrio* species and act though complex signal transduction cascades involving cell density-dependent synthesis, release, and detection of signal molecules called autoinducers [1,2]. AphA and HMR [an abbreviation of high cell density (HCD) master regulator] are the two master QS regulators that are abundantly expressed at low cell density (LCD) and HCD, respectively, and a reciprocally gradient production of these two regulators has been recorded with transition between LCD and HCD [3–6] (see also Figure 1). Notably, HMR has distinct names in different *Vibrio* species, e.g. OpaR in *V. parahaemolyticus* [7], LuxR in *V. harveyi* [8], HapR in *V. cholerae* [9], and SmcR in *V. vulnificus* [10].

*V. parahaemolyticus* is a natural inhabitant of estuarine and marine environments. A small proportion of *V. parahaemolyticus* isolates, which harbor one or more key virulence genes [11,12], are pathogenic to human beings. *V. parahaemolyticus* is a worldwide cause of food-borne gastroenteritis which is usually self-limited and lasts within several days, but severe *V. parahaemolyticus*-caused diseases may occur in persons with weakened immune systems [12,13]. *V. parahaemolyticus* can also cause a skin infection if the bacterium gets in an open sore [12,13].

Type VI secretion system (T6SS) is a bacterial protein injection machinery with roles in virulence, symbiosis, interbacterial interaction, anti-pathogenesis, and environmental stress responses. There are two separate T6SS gene clusters, namely T6SS1 (VP1386-1414) and T6SS2 (VPA1024-1046), in chromosomes I and II of *V. parahaemolyticus*, respectively [14]. T6SS2 harbors 23 consecutive genes that can be assigned into three putative operons, VPA1027-1024, VPA1043-1028, and VPA1044-1046. T6SS1 is present in the majority of clinical isolates but only in a very low fraction of environmental isolates, while T6SS2 is universally present in the *V. parahaemolyticus* populations [15]. T6SS1 is predominantly expressed and active under high-salt marine-like conditions, while elevated secretion of T6SS2 effectors occurs under low salt conditions [16]. T6SS1 rather than T6SS2 has an anti-bacterial activity and, thus, is responsible for interbacterial competition under warm marine conditions, suggesting a role of T6SS1 in enhancing fitness of clinical isolates in marine environments [16]. Both T6SS1 and T6SS2 contribute to adhesion of *V. parahaemolyticus* to host cells [15].

QS-dependent expression of T6SS has be characterized in a bit of bacterial species such as *Vibrio cholerae* [5,11], *V* *. alginolyticus* [17], *Pseudomonas aeruginosa* [4,18], *Yersinia pseudotuberculosis* [19], and *Aeromonas hydrophila* [20]. It has been also previously shown that *V. parahaemolyticus* OpaR is a positive regulator of T6SS2 and a negative regulator of T6SS1 [16,21,22], but detailed mechanism of action of OpaR is unclear. These observations encourage us to hypothesize cell density- and QS-dependent expression of T6SS2 in *V. parahaemolyticus*. This study reported that OpaR bound to the promoter regions of VPA1027-1024, VPA1043-1028, and VPA1044-1046 to stimulate their transcription, while AphA negatively regulated their transcription most likely through acting on OpaR (Figure 1). In addition, this regulation led to a gradient increase of T6SS2 transcription with transition from LCD to HCD (Figure 1).

## Materials and Methods

### Bacterial strains

The wild-type (WT) *V. parahaemolyticus* strain RIMD 2210633 is isolated from a patient with traveler’s diarrhea in Japan in 1996 [14]. The base pairs (bps) 2 to 516 of coding region of *aphA* or the entire coding region of *opaR* were deleted from WT in our previous studies [4,5], generating the *ΔaphA* or *ΔopaR* mutant, respectively. The non-polar deletion of *aphA* or *opaR* was verified in Figure S1. The empty and recombinant pHRP309 plasmids tested were transformed into WT, *ΔaphA*, and *ΔopaR* for LacZ fusion assays (see below). All the primers used in the present work were listed in Table 1.

**Table 1:** Oligonucleotide primers used in this study.

**Target**	**Primers (forward/reverse, 5'–3')**		
**Primer extension**			
*aphA*	/GCTCTTACTGGCGCTTGAG		
*opaR*	/ATCCATTTTCCTTGCCATTTG		
VPA1027	/CTGCATGCTAATCTCCTAGAGC		
VPA1043	/GATTTGAAGCTTTAATTATTAACAT		
VPA1044	/CCGCTATCGCTGCTATTT		
**LacZ fusion**			
VPA1027	GCGCGTCGACTATTACCTTACTTGCCTCTCGG /GCGCGAATTCTGCTTCACGGTCCATTGC		
VPA1043	GCGCGTCGACTTTGTTGATAGGTGGTATTGTG /ATATGAATTCTGAGCGTCCGAAGGTTAC		
VPA1044	GCGCGTCGACGGGACAAAGCAAGCTCATTC /ATATGAATTCAGCGGAGTCTTGTTTATTAACG		
**Protein production**		
*aphA*	AGCGGGATCCATGTCATTACCACACGTAATC /AGCGAAGCTTTTAACCAATCACTTCAAGTTC		
*opaR*	AGCGGGATCCATGGACTCAATTGCAAAGAG /AGCGAAGCTTTTAGTGTTCGCGATTGTAG		
**Complementation of mutant ^#^**		
*aphA*	GATTCTAGAA G G A G G ***AATTCACC***ATGTCATTACCACACGTAATC/ GACAAGCTTTTAACCAATCACTTCAAGTTC		
*opaR*	GATTCTAGAA G G A G G ***AATTCACC***ATGGACTCAATTGCAAAGAG /GACAAGCTTTTAGTGTTCGCGATTGTAG		
**EMSA**		
VPA1027	TATTACCTTACTTGCCTCTCGG/TGCTTCACGGTCCATTGC		
VPA1043	AGCGGAGTCTTGTTTATTAACG/CGAGAAAATCTAACCGAAG		
VPA1044	TTGTGGAAACTCGTTATGG/TTGACGGGTGAAAGTTTGAG		
16S rRNA	GACACGGTCCAGACTCCTAC/GGTGCTTCTTCTGTCGCTAAC		
**DNase I footprinting**		
VPA1027	GAGTTGCTTCATAATAAC/GTTCCGCTGTCGCTTCAC TATTACCTTACTTGCCTCTCGG/CGTCTTACCATTAAGAATTGC		
VPA1043	AGCGGAGTCTTGTTTATTAACG/CGAGAAAATCTAACCGAAG		
VPA1044	TTGTGGAAACTCGTTATGG/TTGACGGGTGAAAGTTTGAG		

#, amplification of the *aphA* or *opaR* coding region together with a ribosome binding site consensus AGGAGG (underlined) and a spacer AATTCACC (bold and italic).

### Bacterial growth conditions

For general *V. parahaemolyticus* cultivation and maintenance, bacteria were cultured in the HI broth [2.5% Bacto heart infusion (BD Bioscience)] or on the HI plate (2.5% Bacto heart infusion, and 1.5% bacteriological grade agar) at 37 °C. For long term storage, bacteria were stored in the HI broth with the addition of 30% glycerol at -85 °C. For the following gene regulation assays, we used a design of two-round precultivation of bacterial cells: firstly, the glyceric stocks of bacteria were inoculated into 15 ml of HI broth for growing at 37 °C with shaking at 200 rpm for 12 to 14 h to enter the stationary growth phase; secondly, the resulting cell cultures were 50-fold diluted into 15 ml of HI broth, and allowed to grow under the above conditions to reach an optical density at 600 nm (OD_600_) of 1.4 to 1.6 (at the mid-exponential growth phases), and then the cell cultures were diluted with the HI broth to an OD_600_ value of 1.4. The precultivated bacterial cells were 1,000-fold diluted into 15 ml of HI broth for a third-round growth under the above conditions for cell harvest at different cell densities.

### RNA isolation and primer extension assay

Total bacterial RNAs were extracted using TRIzol Reagent (Invitrogen) [4,5]. RNA quality was monitored by agarose gel electrophoresis, and RNA quantity was determined by spectrophotometry. For primer extension assay [4,5], an oligonucleotide primer complementary to a portion of RNA transcript of each indicated gene was employed to synthesize cDNAs from RNA templates. Three to 10 µg of total RNA was annealed with 1 pmol of [γ-^32^P] end-labeled reverse primer using a Primer Extension System (Promega) according to the manufacturer’s instructions. For a single target gene, the same amount of total RNAs was used as starting materials to determine its relative mRNA levels in different isogenic strains grown at different cell densities. The same labeled primer was also used for sequencing with an AccuPower & Top DNA Sequencing Kit (Bioneer). The primer extension products and sequencing materials were concentrated and analyzed in a 6% polyacrylamide/8 M urea gel. The result was detected by autoradiography (Kodak film).

### LacZ fusion and β-galactosidase assay

The promoter-proximal DNA region of each indicated gene was amplified by PCR with ExTaq™ DNA polymerase (Takara) using RIMD 2210633 genome DNA as the template. PCR fragments were then cloned between *Sal*I and *EcoR*I sites of low-copy-number transcriptional *lacZ* fusion vector pHRP309 that harbors a gentamicin resistance gene and a promoterless *lacZ* reporter gene [19]. Correct cloning was verified by DNA sequencing. An empty pHRP309 plasmid was also introduced into each strain tested as the negative control. *V. parahaemolyticus* strains transformed with recombinant or empty pHRP309 plasmids were grown as above to measure the β-galactosidase activity in cellular extracts using a β-Galactosidase Enzyme Assay System (Promega) [4,5].

### Preparation of 6× His-tagged OpaR (His-OpaR) and AphA (His-AphA) proteins

The preparation of purified His-OpaR or His-AphA protein was done as described previously [4,5]. The entire coding region of *opaR* or *aphA* of strain RIMD 2210633 was cloned between *BamH*I and *Hind*III sites of plasmid pET28a (Novagen). The recombinant plasmid encoding His-OpaR or His-AphA was transformed into *E. coli* BL21λDE3 cells, and grown in the Luria-Bertani (LB) broth at 37 °C with shaking at 200 rpm for 4 to 5 h. The resulting culture was diluted 1/100 into 200 to 300 ml of fresh LB broth, and grown under the above conditions to an OD_600_ of about 0.5. The culture was shifted to 18 °C for 1 h, and then induced with 1 mM IPTG for 16 to 18 h with shaking at 100 rpm. Cells were collected by centrifugation and frozen at -60 °C. The pellet was resuspended in 10 ml of 50 mM sodium phosphate buffer, pH 7.4, 500 mM NaCl, and 5 mM imidazole. Cells were disrupted using a cell cracker, and the insoluble material was pelleted by centrifugation at 12,000 rpm. The clarified supernatant was applied to a 3 ml Ni-NTA Agarose Column (Qiagen), and the overproduced protein was purified under native conditions. Fractions from a homogenous peak were pooled, and the final preparation was dialyzed against 10 mM Tris HCl, pH 7.4, 10 mM NaCl, 1 mM EDTA, 0.1 mM DTT, and 20% glycerol. The purified protein was stored at -60 °C, and the protein purity was verified by SDS-PAGE.

### Electrophoresis mobility shift assay (EMSA)

The promoter-proximal DNA region of each indicated gene was amplified by PCR. For EMSA [4,5], the 5′ ends of DNA were labeled using [γ-^32^P] ATP and T4 polynucleotide kinase. DNA binding was performed in a 10 µl reaction volume containing binding buffer [1 mM MgCl_2_, 0.5 mM EDTA, 0.5 mM DTT, 50 mM NaCl, 10 mM Tris-HCl (pH 7.5) and 0.05 mg/ml poly-(dI-dC)], labeled DNA (1000 to 2000 c.p.m/µl), and increasing amounts of His-AphA. Three controls were included in each EMSA experiment: 1) cold probe as specific DNA competitor (the same promoter-proximal DNA region unlabeled), 2) negative probe as non-specific DNA competitor (the unlabeled coding region of the 16S rRNA gene), and 3) non-specific protein competitor [rabbit anti-F1-protein polyclonal antibodies]. The F1 protein is the protective antigen from *Yersinia pestis* [23]. After incubation at room temperature for 30 min, the products were loaded onto a native 4% (w/v) polyacrylamide gel, and electrophoresed in 0.5× TBE buffer for about 50 min at 220 V. Radioactive species were detected by autoradiography after exposure to Kodak film at -70 °C.

### DNase I footprinting

For DNase I footprinting [4,5], the promoter-proximal DNA regions with a single ^32^P-labeled end were PCR amplified with either sense or antisense primer being end-labeled. The PCR products were purified using the QiaQuick columns (Qiagen). Increasing amounts of His-AphA or His-OpaR were incubated with the purified, labeled DNA fragment (2 to 5 pmol) for 30 min at room temperature, in a final 10 µl reaction volume containing the binding buffer used in EMSA. Before DNA digestion, 10 µl of Ca^2+^/Mg^2+^ solution (5 mM CaCl_2_ and 10 mM MgCl_2_) was added, followed by incubation for 1 min at room temperature. The optimized RQ1 RNase-Free DNase I (Promega) was then added to the reaction mixture, and the mixture was incubated at room temperature for 40 to 90 s. The reaction was quenched by adding 9 µl of stop solution (200 mM NaCl, 30 mM EDTA, and 1% SDS), followed by incubation for 1 min at room temperature. The partially digested DNA samples were extracted with phenol/chloroform, precipitated with ethanol, and analyzed in 6% polyacrylamide/8 M urea gel. Protected regions were identified by comparison with sequencing ladders. The templates for sequencing were the same as DNA fragments for DNase I footprinting. Radioactive species were detected as above.

### Computational promoter analysis

The 400 bp upstream regions of the genes tested (Table 2) were retrieved from the genome sequence of RIMD 2210633 with the ‘retrieve-seq’ program [18]. The position-specific scoring matrix (PSSM) representing the conserved signals for AphA [5] or OpaR [4] recognition was used for pattern matching within target DNA regions, by using the *matrices-paster* tool [18].

**Table 2:** Prediction of AphA/OpaR box-like sequences within upstream DNA regions

			First gene				AphA box-like sequence									OpaR box-like sequence							
Operon			ID		Name		Position^&^		Sequence					Score	Position^&^		Sequence			Score	
VPA1027-1024			VPA1027		*hcp2*		R-119. . -100	ATACGCTCCTTTATATCTTT					3.98		D-389…- 370	TAATGACATTGTAGACAATA					9.01
															D-87… 68	TTTTGATACATCAATCATTA						8.29
															D-57. . -38	TTCAGATAATTTAATTAATA						9.45
VPA1043-1028			VPA1043		NA		R-196. . -177	ATATCCAACCAGGTTCAAAT					2.51		D-356…- 337	TATTTATAGATTTGTCTTTA				9.33
VPA1044-1046			VPA1044		NA		D-143. . -124	ATATCCAACCAGGTTCAAAT					2.51		D-250. . -231	TATTAACATTAAGATTAATA					9.9

&, ‘D’ indicates the direct sequence while ‘R’ the reverse one; minus numbers denote the nucleotide positions upstream of indicated genes. NA, Not Applicable

### Experimental replicates and statistical methods

For LacZ fusion assays, experiments were performed with at least three independent bacterial cultures, and values were expressed as mean ± standard deviation (SD). Statistical testing of difference was made by Student’s paired *t* test, and a *P* value of <0.01 was taken as significant. For primer extension, EMSA, and footprinting, representative data from at least two independent biological replicates were shown.

## Results

### Predicted AphA/OpaR box-like sequences within T6SS2 gene cluster

The first genes of the three T6SS2 operons VPA1027-1024, VPA1043-1028, VPA1044-1046 were subjective to computational promoter analysis and further gene regulation experiments. The previously characterized PSSMs of AphA [5] and OpaR [4] were used to statistically predict the presence of AphA/OpaR box-like elements within the promoter-proximal regions of the above three ‘first genes’. This analysis generated weight scores for each target promoter, and the higher score values indicated the higher probability of AphA/OpaR-promoter association [17]. When a frequently used score of seven was taken as the cutoff value, OpaR box-like sequences were found for all the three genes, while none of AphA box-like elements could be identified for them (Table 2).

### Negative regulation of T6SS2 genes by AphA

**Figure 2:**
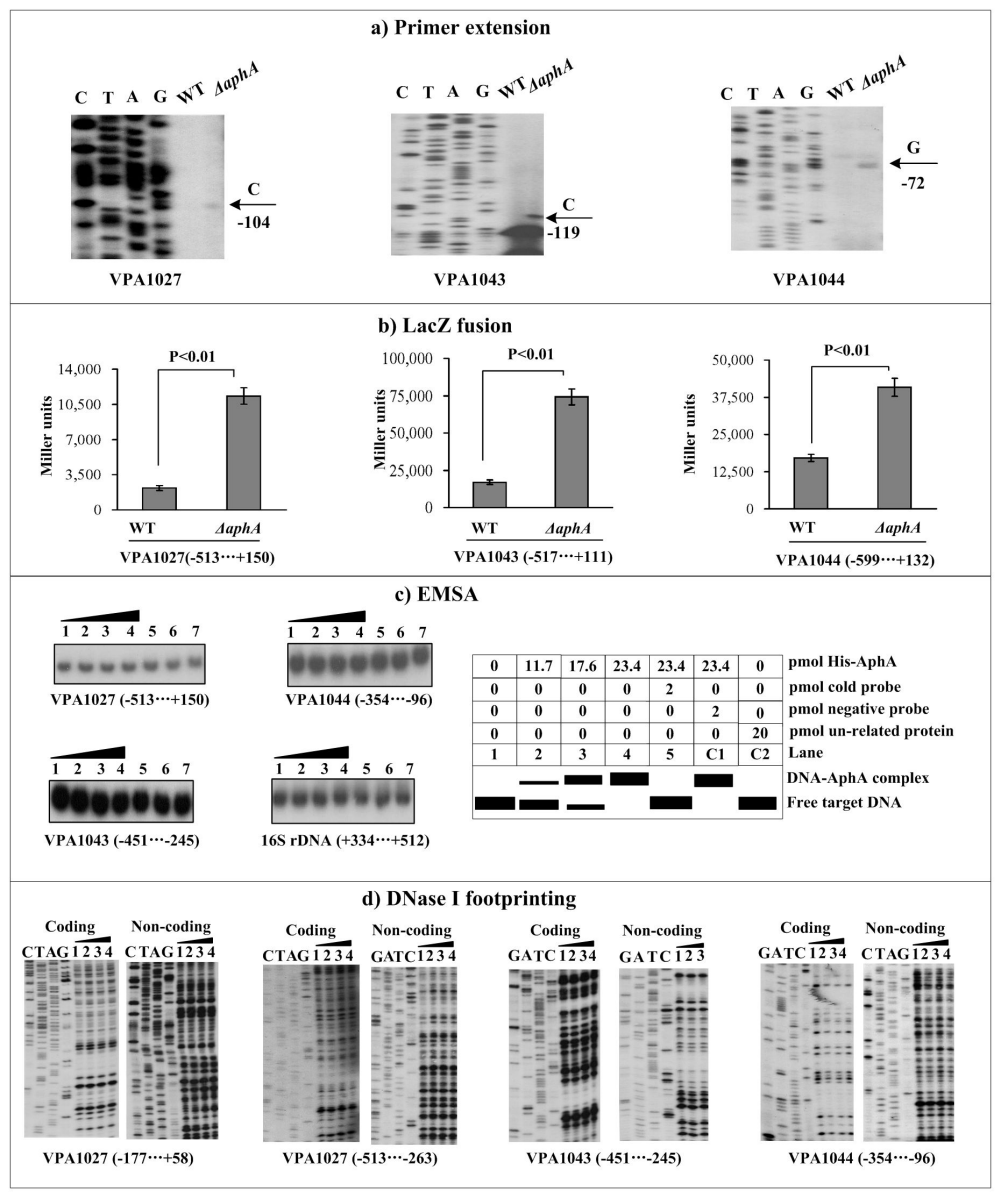
Regulation of T6SS2 genes by AphA. Lanes C, T, A, and G represent the Sanger sequencing reactions. The minus and positive numbers indicated the nucleotide positions upstream and downstream of indicated genes. **a**) **Primer extension**. An oligonucleotide primer was designed to be complementary to the RNA transcript of each gene tested. The primer extension products were analyzed with an 8 M urea-6% acrylamide sequencing gel. The transcriptional start sites were indicated by arrows with nucleotides and positions. **b**) **LacZ fusion**. The target promoter-proximal DNA region was cloned into the *lacZ* transcriptional fusion vector pHRP309 and then transformed into WT or *ΔAphA* to determine the promoter activity, i.e., the β-galactosidase activity (miller units) in the cellular extracts. **c**) **EMSA**. The radioactively labeled promoter-proximal DNA fragments were incubated with increasing amounts of purified His-AphA protein and then subjected to 4% (w/v) polyacrylamide gel electrophoresis. If there was the association of His-AphA and target DNA, the band of free DNA disappeared with increasing amounts of His-AphA, resulting in a retarded DNA band with decreased mobility, which presumably represented the DNA-AphA complex. Shown also was the schematic representation of the EMSA design. **d**) **DNase I footprinting**. Labeled coding or non-coding DNA probes were incubated with increasing amounts of purified His-AphA (Lanes 1, 2, 3, and 4 containing 0, 35.1, 46.8, and 58.5 pmol, respectively) and then subjected to DNase I footprinting assay. The footprint regions were indicated by vertical bars with positions.

For the following primer extension and LacZ fusion assays, bacterial cells were harvested at an OD_600_ value of about 0.2 to simulate the LCD conditions at which AphA was predominantly expressed. The primer extension assay (Figure 2a) indicated that the mRNA levels of all the three genes VPA1027, VPA1043, and VPA1044 evidently enhanced in *ΔahpA* relative to WT. The transcriptional *lacZ* fusion vector that contained the target promoter-proximal DNA region and the promoterless *lacZ* gene was transformed into WT and *ΔaphA* to compare the promoter activities of each of the above three genes in these two strains (Figure 2b). The LacZ fusion experiments disclosed that the promoter activity of each of the above three genes significantly enhanced in *ΔaphA* relative to WT. The promoter-proximal DNA regions of the above three genes were amplified, radioactively labeled, and subjected to EMSA with a purified His-AphA protein (Figure 2c). Negative EMSA results were observed for all the above three genes, but positive results were observed for the positive control gene *opaR* as described previously [5] (data not shown). Further DNase I footprinting experiments (Figure 2d) could not detected footprint for all the above three genes; these were consistent with the EMSA results. Taken together, *V. parahaemolyticus* AphA appears to negatively regulate the transcription of VPA1027-1024, VPA1043-1028, and VPA1044-1046 in an indirect manner.

### Positive regulation of T6SS2 genes by OpaR

**Figure 3:**
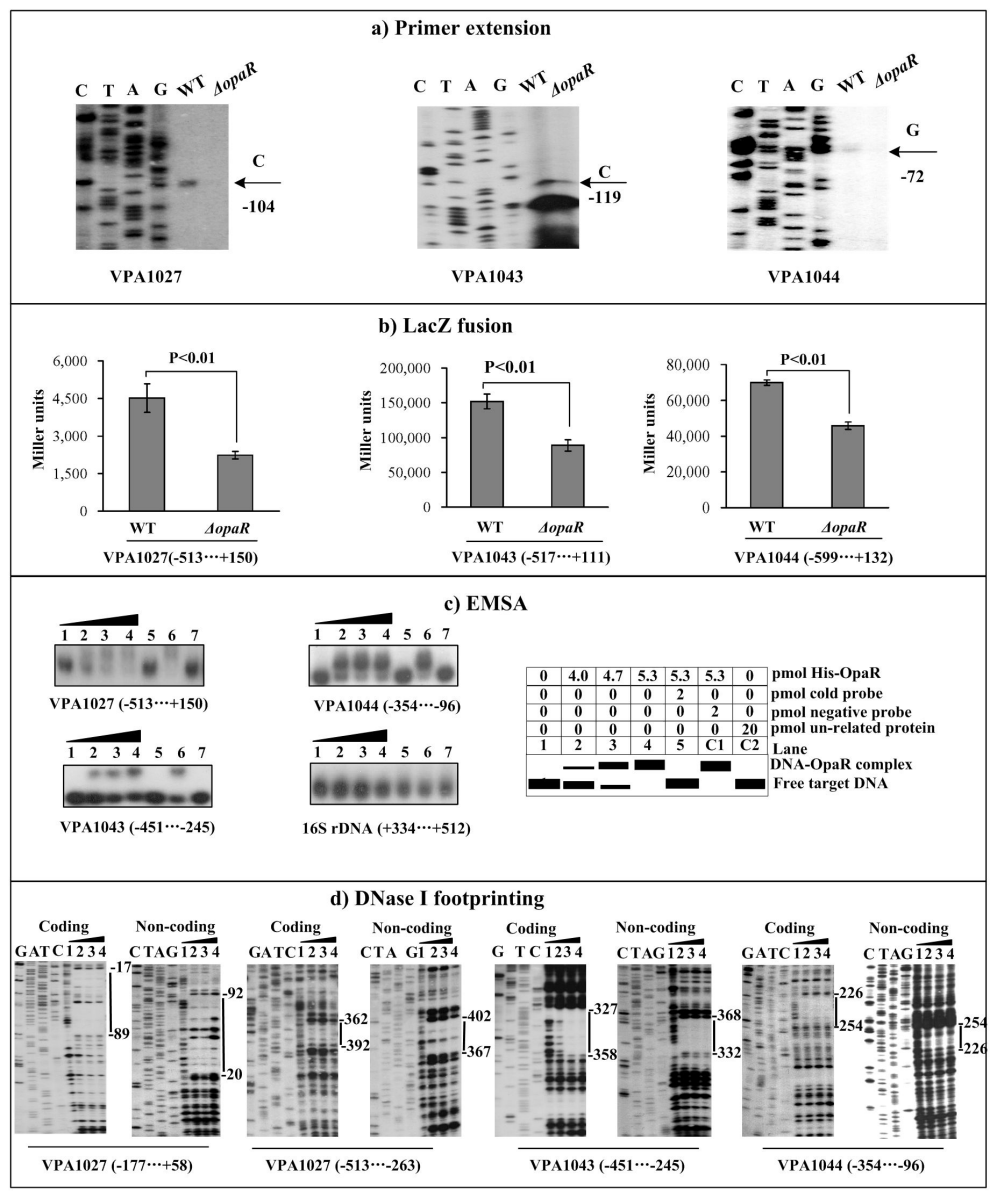
Regulation of T6SS2 genes by OpaR. The primer extension (a), LacZ fusion (b), EMSA (c), DNase I footprinting (d) assays were performed to characterize the regulation of VPA1027-1024, VPA1043-1028, and VPA1044-1046 operons by OpaR. See Figure 2 for detail annotations.

Bacterial cells were harvested at an OD_600_ value of about 1.2 to simulate the HCD conditions at which OpaR was predominantly expressed. Both primer extension (Figure 3a) and LacZ fusion (Figure 3b) assays disclosed that the transcription of the three genes VPA1027, VPA1043, and VPA1044 decreased in *ΔopaR* relative to WT. As determined by EMSA (Figure 3c), a purified His-OpaR protein was able to bind to the upstream DNA fragments of all these three genes in a dose-dependent manner. By using DNase I footprinting (Figure 3d), His-OpaR protected a single DNA region upstream of each of VPA1027, VPA1043, and VPA1044 in a dose-dependent manner, which was consistent with the EMSA results. Each of the footprints detected for VPA1027, VPA1043, and VPA1044 contained one or more OpaR box-like sequences as predicted in Table 2, and was considered as the OpaR site for each target gene. Taken together, *V. parahaemolyticus* OpaR can bind to the promoter regions of VPA1027-1024, VPA1043-1028, and VPA1044-1046 to stimulate their transcription.

### Cell density-dependent transcription of aphA, opaR, and VPA1027

**Figure 4:**
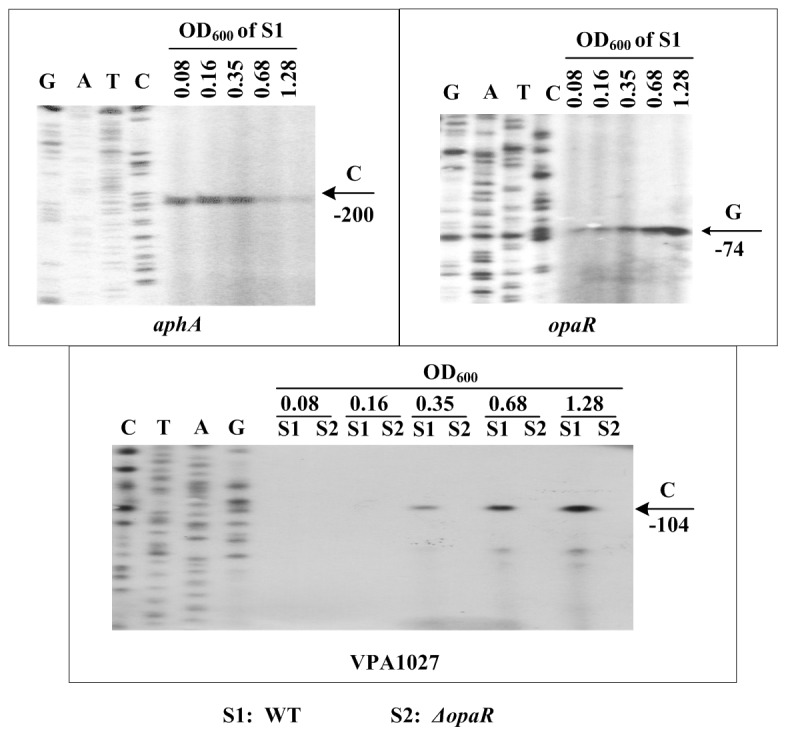
Gene transcription pattern during growth. The bacterial cells were harvested at various OD_600_ values. An oligonucleotide primer was designed to be complementary to the RNA transcript of each gene tested. The primer extension products were analyzed with an 8 M urea-6% acrylamide sequencing gel. Lanes C, T, A, and G represented Sanger sequencing reactions. The transcriptional start sites were indicated by arrows with nucleotides and positions. The minus numbers under the arrows indicated the nucleotide positions upstream of the indicated genes.

The mRNA levels of *aphA*, *opaR*, and VPA1027 (as a representative of T6SS2 genes) were measured in WT grown at various OD_600_ values (i.e. at different cell densities) by the primer extension assay (Figure 4). The *aphA* mRNA levels decreased considerably with the increasing of cell density, whereas the mRNA levels of *opaR* and VPA1027 increased dramatically with increasing of cell density. These results not only confirmed the rationality of cell harvest at an OD_600_ value of about 0.2 or 1.2 for characterizing AphA- or OpaR-mediated gene regulation, respectively, but also indicated the gradual elevation of mRNA levels of T6SS2 genes with transition from LCD to HCD.

## Discussion

OpaR inhibits *V. parahaemolyticus*-induced cytotoxicity to host cells, mostly though acting on type three secretion system 1 (T3SS1) that is the major determinant of cytotoxicity in *V. parahaemolyticus* [24–26]. AphA enhances lethality in mice and cytotoxic activity in *V. parahaemolyticus* [27]. The detailed roles of OpaR and AphA in regulating virulence genes in *V. parahaemolyticus* need to be further elucidated.

As characterized in this study, OpaR and AphA acted as the positive and negative regulators of T6SS2, respectively, leading to a gradient elevation of transcriptional levels of T6SS2 with transition from LCD to HCD (Figure 1). The positive regulation of the three T6SS2 operons VPA1027-1024, VPA1043-1028, and VPA1044-1046 by OpaR achieved through direct association between OpaR and its target promoters. In contrast, AphA negatively regulated T6SS2 genes most likely through acting on OpaR, given the facts that none of the above promoter-proximal regions could be bound by AphA (this study) and that AphA and OpaR acted as transcriptional repressors to interact with each other [4,5]. These observations strongly supported the notion that T6SS2 might play important roles at the middle/late stages of growth/infection.

**Figure 5:**
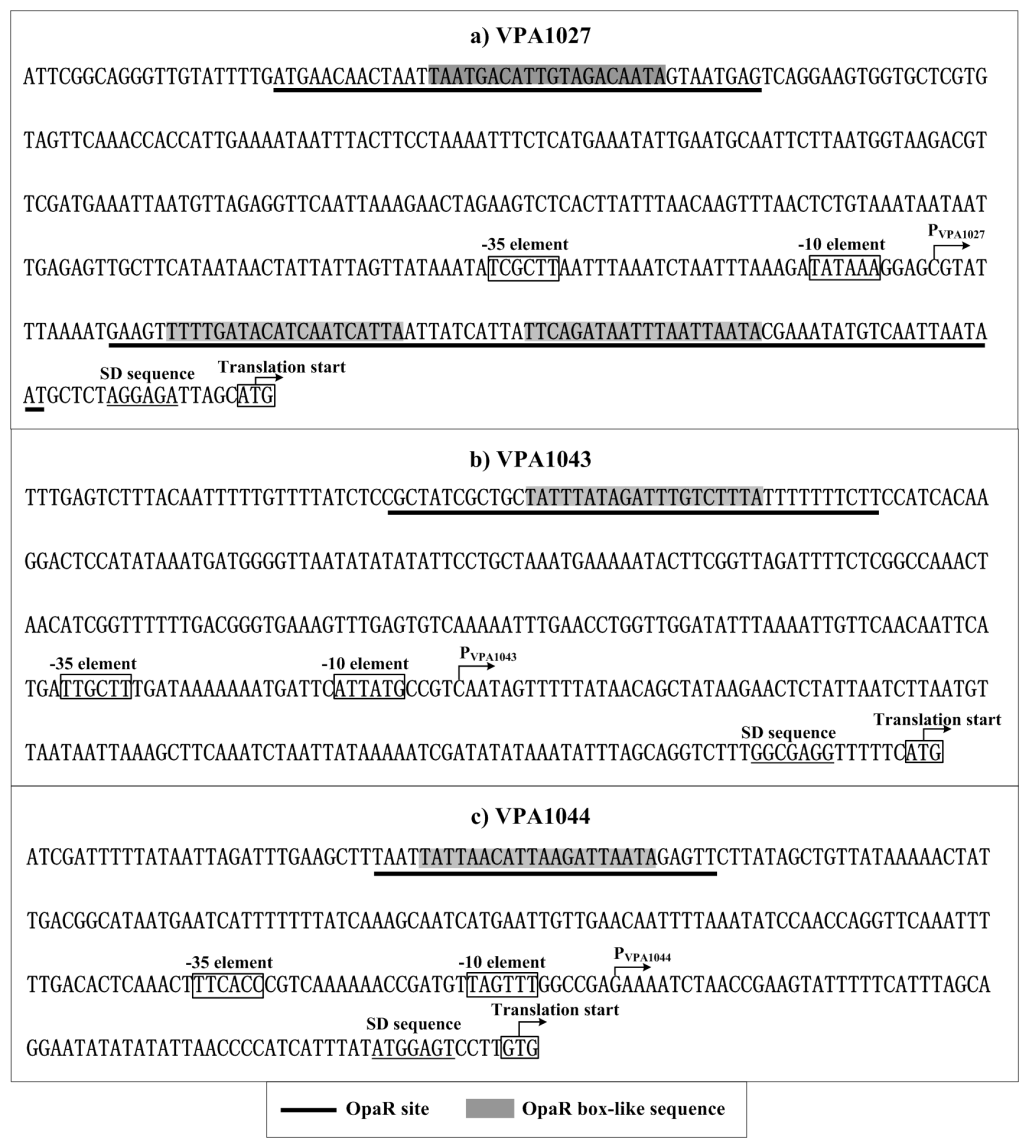
Organization of promoter-proximal DNA regions. The promoter-proximal DNA regions of indicated genes were derived from RIMD 221063.

Collection of data of translation/transcription start sites, core promoter -10 and -35 elements, OpaR sites, OpaR box-like sequences, Shine-Dalgarno (SD) sequences (ribosomal binding sites) enabled us to depict the organization of AphA/OpaR-dependent promoters characterized herein (Figure 5). The OpaR sites for VPA1043-1028 and VPA1044-1046 were upstream of promoter -35 elements and, thus, both of these OpaR-dependent promoters might have a class I transcriptional stimulation that depends on the RNAP α subunit C-terminal domain for function [28]. Binding of OpaR to the upstream region of VPA1027-1024 was highly unusual, because two different OpaR sites, upstream and downstream of the -35 and -10 core promoter regions, respectively, were identified.

Three additional gene loci have been shown to be positively regulated by HMRs in a direct manner in different *Vibrio* species. *V. harveyi* LuxR binds to the upstream DNA region of the luminescence operon *luxCDABEGH* to stimulate its transcription [29]. *Vibrio cholerae* HapR [30] and *V. vulnificus* SmcR [31] stimulate the metalloprotease genes *hapA* and *vvpE*, respectively. Given the fact that the reciprocally gradient production of AphA and HMR is a conserved mechanism employed by multiple *Vibrio* species [3–6], the above three HMR-stimulated gene loci would show an elevated production in a gradient manner with transition from LCD to HCD.

## Supporting Information

Figure S1 **Primer extension assay for validation of non-polar deletion of *opaR* or *aphA*.** For complementation of *ΔopaR* or *ΔaphA*, a PCR-generated DNA fragment composed of the entire coding region of *opaR* or *aphA*, respectively, together with a upstream synthetic ribosome binding site (Table 1), was cloned into between the *Xba*I and *HinD*III sites of pBAD33 vector [42] harboring an arabinose P_BAD_ promoter and a chloramphenicol resistance gene. The resulting recombinant plasmid pBAD33-*opaR* or pBAD33-*aphA*, respectively, was then introduced into *ΔopaR* or *ΔaphA* through electrotransformation, yielding the complemented mutant strain *ΔopaR*/pBAD33-*opaR* or *ΔaphA/*pBAD33-*aphA*, respectively. In addition, the empty vector pBAD33 was introduced into WT or *ΔopaR* or *ΔaphA* to generate the strain named WT/pBAD33 or *ΔopaR*/pBAD33 or *ΔaphA*/pBAD33, respectively. Bacteria were cultivated as described in the main text, with the modification that 5 µg/ml chloramphenicol and 0.1% arabinose were added in cell cultures. The primer extension experiments were subsequently done to determine the relative mRNA levels of VPA1027 in WT/pBDA33, *ΔopaR*/pBDA33, *ΔopaR/*pBDA33-*opaR*, *ΔaphA*/pBDA33, and *ΔaphA/*pBDA33-*aphA*. The mRNA level was significantly repressed in *ΔopaR*/pBDA33 relative to either WT/pBDA33 or *ΔopaR/*pBDA33-*opaR*, and, yet, it was significantly enhanced in *ΔaphA*/pBDA33 compared to either WT/pBDA33 or *ΔaphA/*pBDA33-*aphA*. These results confirmed that the *opaR* or *aphA* deletion was nonpolar.
(TIF)
