# Supplementary figures and images for: Cell Density- and Quorum Sensing-Dependent Expression of Type VI Secretion System 2 in Vibrio parahaemolyticus
Source: PLoS One. 2013 Aug 15;8(8):e73363. doi: 10.1371/journal.pone.0073363 (PMC3744643; doi:10.1371/journal.pone.0073363)

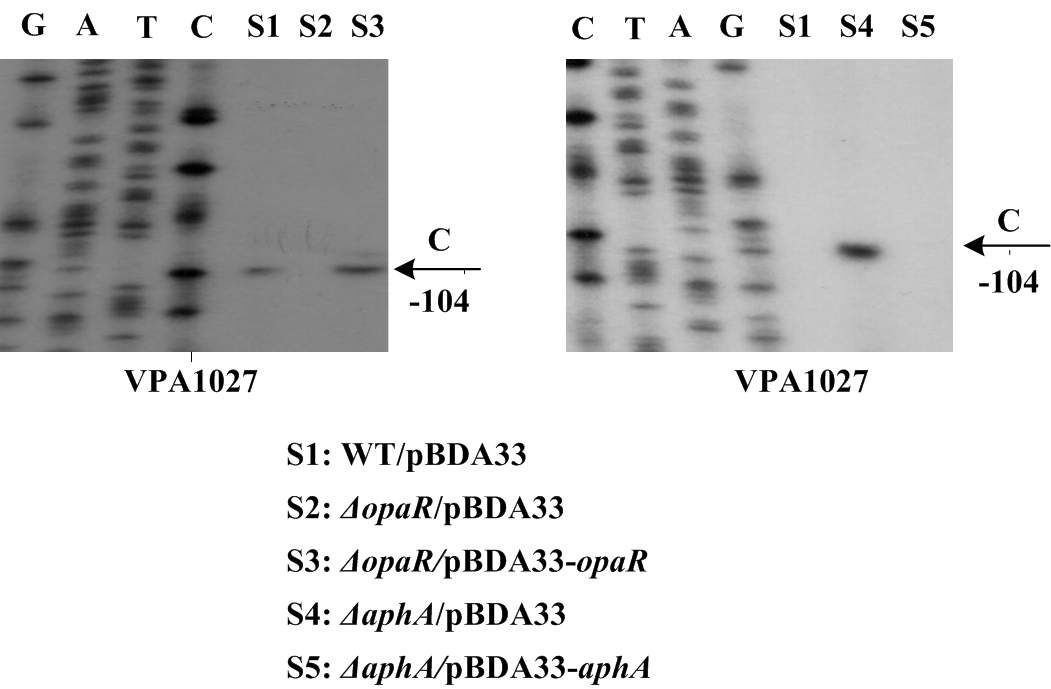

Supplement: Figure S1 — Primer extension assay for validation of non-polar deletion of opaR or aphA. For complementation of ΔopaR or ΔaphA, a PCR-generated DNA fragment composed of the entire coding region of opaR or aphA, respectively, together with a upstream synthetic ribosome binding site (Table 1), was cloned into between the XbaI and HinDIII sites of pBAD33 vector [42] harboring an arabinose PBAD promoter and a chloramphenicol resistance gene. The resulting recombinant plasmid pBAD33-opaR or pBAD33-aphA, respectively, was then introduced into ΔopaR or ΔaphA through electrotransformation, yielding the complemented mutant strain ΔopaR/pBAD33-opaR or ΔaphA/pBAD33-aphA, respectively. In addition, the empty vector pBAD33 was introduced into WT or ΔopaR or ΔaphA to generate the strain named WT/pBAD33 or ΔopaR/pBAD33 or ΔaphA/pBAD33, respectively. Bacteria were cultivated as described in the main text, with the modification that 5 µg/ml chloramphenicol and 0.1% arabinose were added in cell cultures. The primer extension experiments were subsequently done to determine the relative mRNA levels of VPA1027 in WT/pBDA33, ΔopaR/pBDA33, ΔopaR/pBDA33-opaR, ΔaphA/pBDA33, and ΔaphA/pBDA33-aphA. The mRNA level was significantly repressed in ΔopaR/pBDA33 relative to either WT/pBDA33 or ΔopaR/pBDA33-opaR, and, yet, it was significantly enhanced in ΔaphA/pBDA33 compared to either WT/pBDA33 or ΔaphA/pBDA33-aphA. These results confirmed that the opaR or aphA deletion was nonpolar. (TIF) [file pone.0073363.s001.tif]
